# Information Processing Speed and 8-Year Mortality Among Community-Dwelling Elderly Japanese

**DOI:** 10.2188/jea.JE20120210

**Published:** 2014-01-05

**Authors:** Hajime Iwasa, Ichiro Kai, Yuko Yoshida, Takao Suzuki, Hunkyung Kim, Hideyo Yoshida

**Affiliations:** 1 Fukushima Medical University School of Medicine, Fukushima, Japan; 1 福島県立医科大学医学部; 2 Graduate School of Medicine, The University of Tokyo, Tokyo, Japan; 2 東京大学大学院医学系研究科; 3 Tokyo Metropolitan Institute of Gerontology, Tokyo, Japan; 3 東京都健康長寿医療センター; 4 National Center for Geriatrics and Gerontology, Obu, Aichi, Japan; 4 国立長寿医療研究センター

**Keywords:** all-cause mortality, cognition, community elderly, information processing speed

## Abstract

**Background:**

Cognitive function is an important contributor to health among elderly adults. One reliable measure of cognitive functioning is information processing speed, which can predict incident dementia and is longitudinally related to the incidence of functional dependence. Few studies have examined the association between information processing speed and mortality. This 8-year prospective cohort study design with mortality surveillance examined the longitudinal relationship between information processing speed and all-cause mortality among community-dwelling elderly Japanese.

**Methods:**

A total of 440 men and 371 women aged 70 years or older participated in this study. The Digit Symbol Substitution Test (DSST) was used to assess information processing speed. DSST score was used as an independent variable, and age, sex, education level, depressive symptoms, chronic disease, sensory deficit, instrumental activities of daily living, walking speed, and cognitive impairment were used as covariates.

**Results:**

During the follow-up period, 182 participants (133 men and 49 women) died. A multivariate Cox proportional hazards model showed that lower DSST score was associated with increased risk of mortality (hazard ratio [HR] = 1.62, 95% CI = 0.97–2.72; HR = 1.73, 95% CI = 1.05–2.87; and HR = 2.55, 95% CI = 1.51–4.29, for the third, second, and first quartiles of DSST score, respectively).

**Conclusions:**

Slower information processing speed was associated with shorter survival among elderly Japanese.

## Abstract (ja)

**〔背景〕:**

認知機能は高齢期の健康維持に重要な影響を及ぼす。認知機能のうち情報処理速度は、認知症発症を予測すること、生活機能の低下と関連することが報告されている。しかし情報処理速度と生命予後の関連はこれまでにあまり報告されていない。本研究では、日本の地域高齢者を対象とした8年間の前向きコホート研究デザインにより、情報処理速度と総死亡の関連を検討した。

**〔方法〕:**

70歳以上の高齢者男性440人と女性371人が本研究に参加した。情報処理速度は符号問題（Digit symbol substitution test)により測定した。符号問題得点を独立変数として、年齢、性別、教育歴、抑うつ傾向、生活習慣病、感覚機能障害、手段的自立、歩行速度、認知障害を共変量として分析に使用した。

**〔結果〕:**

追跡期間中に182人（男性133人、女性49人）が死亡した。Coxの比例ハザードモデルの結果、符号問題得点が低いほど死亡リスクが高いことが明らかとなった（第3四分位群におけるハザード比：1.62、95%信頼区間：0.97～2.72; 第2四分位群におけるハザード比：1.73、95%信頼区間：1.05～2.87; 第1四分位群におけるハザード比：2.55、95% 信頼区間：1.51～4.29）。

**〔結論〕:**

本研究より、情報処理速度が遅い高齢者は生命予後が不良であることが示唆された。

## INTRODUCTION

Cognitive function is an important contributor to health among elderly adults. Recent studies have identified a longitudinal association between cognitive function and mortality among older adults.^1^^,^^2^ One reliable measure of cognitive functioning is information processing speed, which refers to how quickly an individual can accurately process new input from the environment and retrieve stored information from memory.^3^ It can be assessed objectively by measuring reaction time^4^ or by using tests such as the Digit Symbol Substitution Test (DSST).^5^ Information processing speed is closely correlated with chronologic age.^6^ Slow processing speed predicts incident dementia^7^ and is longitudinally related to the incidence of functional dependence in activities of daily living (ADL) and instrumental ADL (IADL).^8^ In addition, recent studies reported that information processing speed among elderly adults can be increased by interventions that improve cognitive functioning.^9^^,^^10^ Thus, because information processing speed has a central role in cognitive aging, is closely associated with incidence of functional dependence, and is responsive to intervention strategies, further study of its relationship to mortality could aid development of longevity-promoting strategies.

Although a few studies have assessed the relationship between information processing speed and all-cause mortality among elderly adults,^2^^,^^11^^,^^12^ the mechanisms underlying this relationship remain unclear. Various factors such as physical health status and cognitive impairment are thought to influence this relationship. Cognitive performance among elderly adults is reported to be adversely affected by physical health problems^13^ such as functional disability, hearing loss, and chronic disease, all of which are closely related to mortality.^14^^–^^16^ Thus, individuals with slower information processing speed may be more likely to have shorter life spans because of their poor physical health. In the present study, we adjusted our statistical model for physical health status—including chronic disease, sensory deficit, IADL, and walking speed—to determine if the relationship between information processing speed and all-cause mortality is independent of physical health status.

Regarding the effect of cognitive impairment on the relationship between information processing speed and all-cause mortality, elderly individuals with early dementia were reported to have slower information processing speed.^7^ Additionally, elderly adults with dementia have shorter life spans than cognitively intact individuals.^17^ Thus, individuals with slower processing speed may have shorter life spans, due to dementia. We also performed a statistical adjustment for cognitive impairment to determine if the relationship between information processing speed and all-cause mortality is independent of cognitive impairment.

In this study, we examined the longitudinal relationship between information processing speed (as measured by the DSST) and all-cause mortality among Japanese community-dwelling elderly adults. Additionally, we sought to determine if the longitudinal relationship was independent of physical health status and cognitive impairment.

## METHODS

### Participants

The data for the present study were collected during mass health checkups for community-dwelling older adults (*Otasha-Kenshin*),^18^^,^^19^ which were conducted by the Tokyo Metropolitan Institute of Gerontology. The Japanese term *Otasha-Kenshin* translates as “health checkups for successful aging.” The study was conducted in Itabashi ward, in northern Tokyo, Japan. The Itabashi ward authorities granted access to the registration files of municipal residents. At the baseline, participants took part in a face-to-face interview with trained research assistants. The study was approved by the Ethics Committee of the Tokyo Metropolitan Institute of Gerontology. The study was described to all participants, who were advised that their participation would be entirely voluntary, that they could withdraw from the study at any time, and they would not be disadvantaged in any way if they chose to withdraw or not participate. As of 2002, a sample of 1945 residents (age 70–84 years) was randomly obtained from the registration files of municipal resident, and we acquired 847 complete datasets (43.5% participation) for the baseline survey.

**Figure 1.**
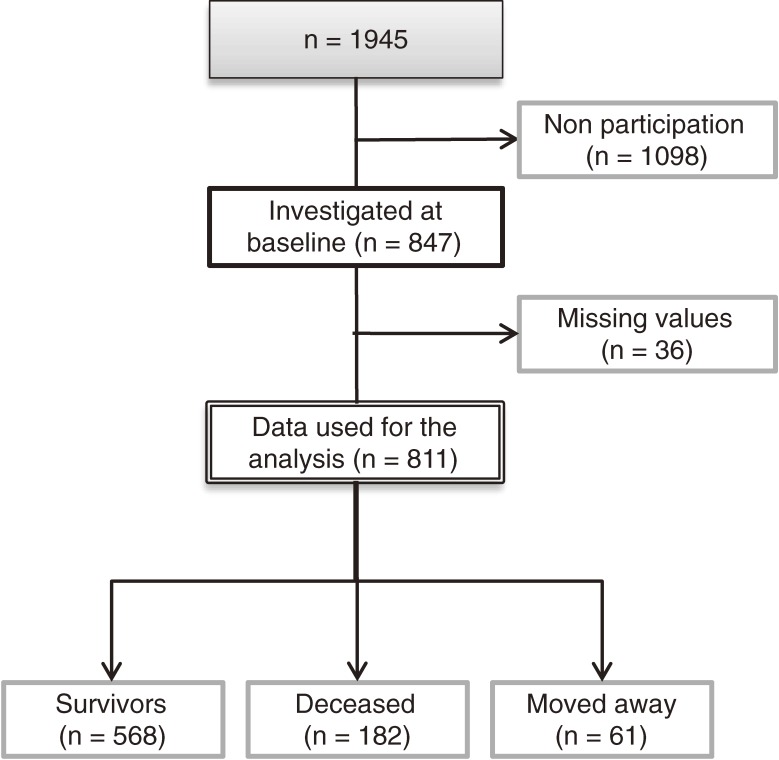
Study flow diagram.

Of those who participated in the baseline survey, 36 were excluded from the analysis because they had missing DSST scores. Thus, 811 participants (440 men and 371 women; mean age at baseline, 76.1 ± 3.6 years) with complete datasets were included, and their data were used for the 8-year mortality surveillance (Figure 1).

### Mortality follow-up

The survey was completed at the end of 2002, and the date of the survey was defined as the baseline for the follow-up period in the present study. Thus, we carried out the 8-year mortality surveillance from the date of the survey to January 1, 2011. Current residency in Itabashi ward on January 1, 2011 was determined using the municipal resident registration files for Itabashi ward. The dates on which residents moved away or died were identified from the registration files and used to calculate survival times. The certifications and dates of all decedents and those moving away were obtained from the Itabashi ward authorities. The dependent variable in the analyses was survival time, calculated as the number of days between the baseline and the date of death or censoring (including survivors and dropouts due to migration from Itabashi ward). Survivors were censored on January 1, 2011. Dropouts were censored on the date of migration from Itabashi ward.

### Measurement of information processing speed

We used the DSST^5^ to assess information processing speed among elderly adults. The DSST is a paper-and-pencil task. The participants receive a test sheet paper and are asked to do a timed translation of numbers to symbols, using a key given at the top of the test page, and write as many symbols as possible into the empty boxes below each digit. The test is scored as the number of correct translations completed within 90 seconds, with a potential range of 0 to 93. We divided the participants into 4 groups using quartiles of the DSST score.

### Other measurements

Data for baseline characteristics were used as covariates in the analysis of the association between cognitive performance and mortality and to describe the characteristics of the study participants. Data for age, sex, education level, chronic disease, sensory deficit, depressive symptoms,^20^ IADL (measured according to the Tokyo Metropolitan Institute of Gerontology Index of Competence^21^), self-rated health, walking speed, and Mini-Mental State Examination (MMSE^22^) score were included. Chronic disease was self-reported by the participants and was defined as having at least 1 of the following diseases: stroke, heart disease, or diabetes mellitus. Sensory deficit was self-reported by the participants and was defined as experiencing at least 1 of the following: hearing loss or eyesight problems. To assess IADL, participants were asked to assess whether they were independent with respect to the 5 daily IADL tasks (eg, using public transportation and preparing meals).^21^ Higher scores reflect a higher level of functioning in IADL. In this study, a cut-off score of 4/5 (ie, a score of ≤4 was classified as IADL-dependent) was used to determine whether participants were dependent with respect to IADL.^23^ To test usual walking speed, participants walked at their usual pace along a straight 11-meter walkway on a flat floor. A stopwatch was used to measure the time taken to walk 5 meters, from the time the foot touched the ground after the 3-meter line to when the foot touched the ground after the 8-meter mark.^24^ The MMSE was used to discriminate participants who had a possible cognitive impairment, using a cutoff score of 23/24, meaning that participants with a score of 0 to 23 points were classified as possibly cognitively impaired.^13^

### Statistical analysis

We used Cox proportional hazards models to test longitudinal relationships between DSST performance and all-cause mortality. In model 1, we adjusted for age, sex, education level, and depressive symptoms at baseline. Model 2 was adjusted for the covariates in model 1 plus chronic disease, sensory deficit, IADL, and walking speed at baseline. In model 3, we adjusted for the covariates in model 2 plus cognitive impairment at baseline. All statistical procedures were performed using SPSS for Windows (version 17.0; SPSS, Inc., Chicago, IL, USA).

## RESULTS

During the 8-year follow-up of the 811 adults, 182 (133 men and 49 women) died and 61 (30 men and 31 women) moved to a different region of Japan and were lost to follow-up.

Table 1 shows the characteristics of the members of the follow-up cohort, collected in 2002, including age, sex, education level, depressive symptoms, chronic disease, sensory deficit, IADL, walking speed, cognitive impairment, DSST scores, and self-rated health.

**Table 1.** Characteristics of study participants (*N* = 811)

Age, mean ± SD (years)	76.1 ± 3.6
Sex (female), *n* (%)	371 (45.7)
Years of education, mean ± SD	10.7 ± 3.1
Depressive symptoms, *n* (%)	19 (2.3)
Chronic disease^a^, *n* (%)	308 (38.0)
Sensory deficit, *n* (%)	99 (12.2)
Instrumental activities of daily living (dependent), *n* (%)	123 (15.2)
Walking speed, mean ± SD (m/s)	1.2 ± 0.3
Cognitive impairment^b^, *n* (%)	46 (5.7)
Digit Symbol Substitution Test score, mean ± SD	35.2 ± 10.9
Self-rated health (fair/poor), *n* (%)	167 (20.6)

^a^Chronic disease was defined as ≥1 of the following diseases: stroke, heart disease, or diabetes mellitus.

^b^A cutoff score of 23/24 on the Mini-Mental State Examination was used to discriminate participants with possible cognitive impairment.

**Figure 2.**
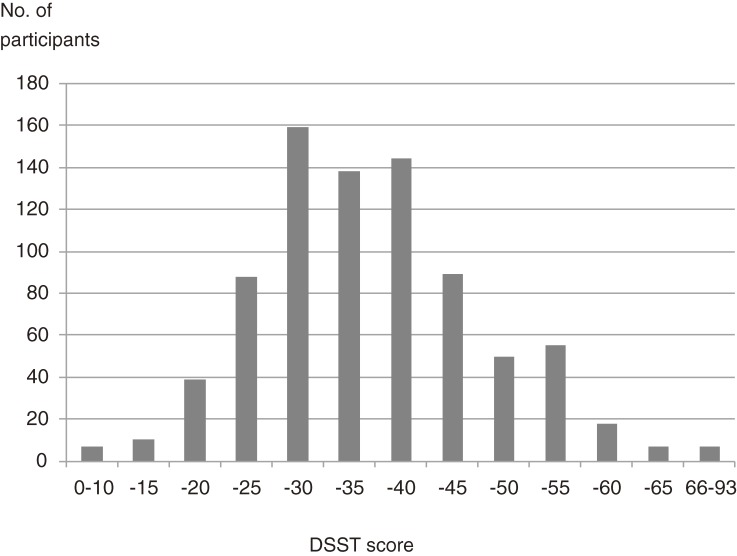
Distribution of DSST scores at baseline.

Figure 2 shows the distribution of DSST scores at baseline. The mean score was 35.2 ± 10.9 (range: 5 to 73).

**Figure 3.**
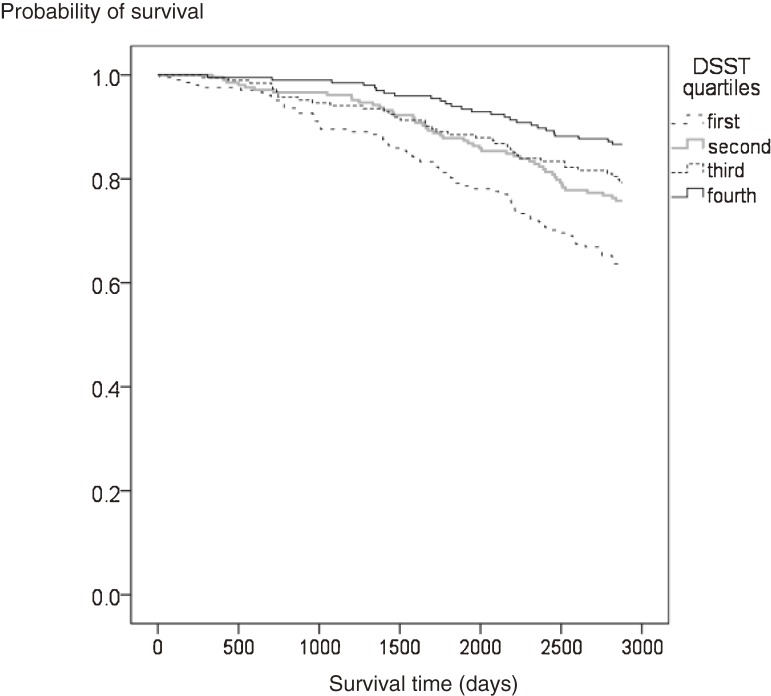
Unadjusted Kaplan–Meier survival curves for all-cause mortality according to DSST quartile at baseline, during an 8-year follow-up period. Mortality risk significantly differed according to DSST quartile (log-rank test: *P* < 0.001). The vertical axis indicates survival probability; the horizontal axis indicates survival time (days).

Figure 3 shows the Kaplan–Meier survival curves for mortality according to quartile of DSST score. Mortality risk was significantly higher among lower-functioning individuals than among higher-functioning individuals (log-rank test: *P* < 0.001).

Table 2 shows the association between DSST and mortality. By DSST quartile, the mean scores ± SD (score range) were 50.1 ± 6.3 (43–73), 38.1 ± 2.1 (35–42), 30.9 ± 1.9 (28–34), and 22.4 ± 4.6 (5–27), for the fourth, third, second, and first quartile quartiles, respectively.

**Table 2.** Adjusted hazard ratios (HRs) for all-cause mortality according to quartile of DSST score (*N* = 811)

Quartile	No.	Deceased (%)	Mean ± SD (range)	Model 1^a^		Model 2^b^		Model 3^c^	
		
				HR (95% CI)	*P*	HR (95% CI)	*P*	HR (95% CI)	*P*
Fourth (reference)	200	26 (13.0)	50.1 ± 6.3 (43–73)	1		1		1	
Third	194	37 (19.1)	38.1 ± 2.1 (35–42)	1.84 (1.11–3.06)	0.018	1.59 (0.96–2.67)	0.073	1.62 (0.97–2.72)	0.068
Second	210	49 (23.3)	30.9 ± 1.9 (28–34)	2.02 (1.24–3.30)	0.005	1.74 (1.06–2.86)	0.029	1.73 (1.05–2.87)	0.033
First	207	70 (33.8)	22.4 ± 4.6 (5–27)	3.42 (2.09–5.58)	<0.001	2.76 (1.66–4.56)	<0.001	2.55 (1.51–4.29)	<0.001

^a^Adjusted for baseline characteristics, including age, sex, education level, and depressive symptoms.

^b^Adjusted for covariates in Model 1 plus chronic disease, sensory deficit, instrumental activities of daily living, and walking speed at baseline.

^c^Adjusted for covariates in Model 2 plus cognitive impairment at baseline.

In multivariate Cox regression analysis adjusted for the above-mentioned potential confounders, lower DSST score was associated with increased mortality risk in model 1 (hazard ratio [HR] = 1.84, 95% CI = 1.11–3.06; HR = 2.02, 95% CI = 1.24–3.30; HR = 3.42, 95% CI = 2.09–5.58, for the third, second, and first quartiles, respectively). In model 2, lower DSST score was associated with increased mortality risk (HR = 1.59, 95% CI = 0.96–2.67; HR = 1.74, 95% CI = 1.06–2.86; HR = 2.76, 95% CI = 1.66–4.56, for the third, second, and first quartiles, respectively). In model 3, as well, lower DSST score was associated with increased mortality risk (HR = 1.62, 95% CI = 0.97–2.72; HR = 1.73, 95% CI = 1.05–2.87; HR = 2.55, 95% CI = 1.51–4.29, for the third, second, and first quartiles, respectively).

## DISCUSSION

In this study, we examined the relationship between information processing speed (as measured by DSST) and all-cause mortality among community-dwelling elderly Japanese during an 8-year follow-up period. Our results indicate that information processing speed at baseline predicted 8-year mortality. As in previous reports,^2^^,^^11^^,^^12^ our findings suggest that slow information processing speed is a reliable predictor of mortality among community-dwelling older adults.

We additionally sought to understand and explain the mechanisms by which information processing speed is related to all-cause mortality. Two potential mechanisms were investigated. First, because of the possibility that individuals who have slower information processing speeds have shorter expected life spans as a consequence of poorer physical health,^13^^–^^16^ we used multivariate analysis (model 2) to examine whether the relationship between information processing and mortality was independent of physical health status. In this model, we adjusted for confounders indicative of poor physical health, including presence of chronic disease, quality of sensory function, IADL, and walking speed. Multivariate analysis showed that DSST score was associated with mortality even after adjustment for these confounders. We therefore conclude that there is an independent relationship between information processing speed and mortality, regardless of physical health status.

Second, we examined if cognitive impairment explains the relationship between information processing speed and mortality. Older individuals with early dementia exhibit slower information processing speed.^7^ Additionally, older individuals with dementia have shorter life spans than those who are cognitively intact,^17^ which suggests that individuals with slower processing speeds may have shorter life spans because of cognitive impairment. To determine whether the relationship between information processing and mortality is independent of cognitive impairment, we performed additional multivariate analysis (model 3) in which we adjusted for cognitive impairment (defined as an MMSE score of ≤23). We found that DSST score was associated with mortality even after adjustment for this potential confounder, suggesting that the relationship between information processing speed and mortality is independent of cognitive impairment. Nevertheless, because dementia was not diagnosed by a specialist in the present study, we cannot eliminate the possibility that participants with dementia were accidentally included in the group with impaired DSST performance.

Although multivariate analysis largely excluded the possibility of confounding by physical health status and cognitive impairment, it is possible that the higher mortality among individuals with slower information processing speeds can be explained by mild cognitive impairment (MCI) in the study population.^25^ As compared with cognitively normal older adults, individuals with MCI reportedly exhibit slower processing speeds in performance-based measures of everyday functional activities.^26^ Because individuals with MCI are prone to develop dementia,^27^ they may be indirectly predisposed toward lower life expectancies. Additionally, recent studies have found that MCI may be a direct risk factor for shorter life spans,^28^ perhaps because individuals with MCI tend to have diminished capabilities for performing everyday tasks (such as taking prescribed medications regularly and keeping medical appointments)^29^ and lower levels of health literacy,^30^ both of which are critical in maintaining health. Although we conducted additional analysis to exclude cognitive impairment (model 3), recent studies have reported that MMSE scores alone may be insufficient for identifying individuals with MCI.^31^ Accordingly, some individuals who exhibited slower processing speeds in this study may have had MCI. Future studies should exclude individuals with MCI, perhaps by using tools that discriminate MCI, such as the Montreal Cognitive Assessment.^32^ Excluding persons with MCI would allow a more direct examination of the relationship between information processing speed and mortality among elderly adults.

Recent studies have reported that interventions to improve information processing speed in elderly adults also transfer to activities instrumental to daily living, such as managing money, preparing meals, and driving.^9^^,^^10^ These studies also suggest that information processing speed is a fundamental function that is closely associated with everyday activities in elderly adults, and that it is amenable to intervention strategies.^9^^,^^10^ Therefore, early detection and treatment of older adults who exhibit slower information processing speed can improve cognitive performance in daily life and may contribute to the development of longevity-promoting strategies.

The representativeness of our study sample could potentially limit the external validity of our findings in 2 ways. First, the characteristics of the study participants were somewhat different from those of the general population. Specifically, the proportion of men in this study was relatively high and study participants were, on average, more educated than the general population (Itabashi ward, 2002; Cabinet office, Government of Japan, 1995). In Japan, education level is higher among elderly men than among elderly women.^33^ Individuals who have higher levels of education are more likely to have good cognitive function during old age.^13^ Therefore, the level of cognitive function in our study sample may be higher than that in the general population. Second, the participation rate at baseline (43.5%) was relatively low because we obtained our data by administering mass health checkups. It has been reported that participants in mass checkups are generally younger than nonparticipants and have higher education levels, fewer chronic diseases, less extensive histories of hospitalization, higher self-rated levels of health, better IADL scores, and better subjective well-being.^18^ Therefore, as a consequence of self-selection bias, it is likely that the participants in our study had different health characteristics than those of nonparticipants.^18^^,^^34^ To improve the representativeness of the study sample, future research should focus on increasing the rate of participation at baseline, which would limit the potential for self-selection bias. We suggest 2 principal methods of increasing baseline participation rates. First, the purpose of the survey should be explained in plain language to family members of potential participants, in addition to the potential participants themselves. This would allow older individuals to arrive at decisions concerning participation in full consultation with their families. Second, it may be necessary to transport participants to and from the checkups, so individuals who are physically frail can take part in the survey without inconvenience. However, this option might increase overall study costs.

For reasons of practicality, several covariates in our study were self-reported by the participants, namely, those related to history of chronic disease (stroke, heart disease, diabetes mellitus) and sensory deficits (hearing loss, eyesight problems). Self-reported evaluations of health status could be less accurate than objective evaluations, which might be obtained from medical records and clinical examinations. Therefore, future studies of the relationship between cognition and mortality should rely on more-objective measures of these covariates, which could eliminate any biases due to self-reporting and increase the precision of recorded data.

In conclusion, we examined the relationship between information processing speed and all-cause mortality among community-dwelling elderly adults in Japan and found that information processing speed (assessed using the DSST) predicted mortality. This suggests that older individuals with slower information processing speed are more likely to have shorter life spans as compared with higher-functioning elderly. Our results may help facilitate development of longevity-promoting strategies and underscore the importance of early detection and treatment of cognitive decline among elderly adults.

## ONLINE ONLY MATERIALS

Abstract in Japanese.
