# Supplementary material for: Information Processing Speed and 8-Year Mortality Among Community-Dwelling Elderly Japanese
Source: J Epidemiol. 2014 Jan 5;24(1):52–9. doi: 10.2188/jea.JE20120210 (PMC3872525; doi:10.2188/jea.JE20120210)
Supplement: Abstract in Japanese. [file je-24-052-s001.pdf]

日本の地域高齢者における情報処理速度と8年間の生命予後の関連

岩佐一[1,2,3]、甲斐一郎[2]、吉田祐子[3]、鈴木隆雄[4]、金憲経[3]、吉田英世[3]

[1]福島県立医科大学医学部

[2]東京大学大学院医学系研究科

[3]東京都健康長寿医療センター

[4]国立長寿医療研究センター

〔背景〕認知機能は高齢期の健康維持に重要な影響を及ぼす。認知機能のうち情報処理速度は、認知症発症を予測すること、生活機能の低下と関連することが報告されている。しかし情報処理速度と生命予後の関連はこれまでにあまり報告されていない。本研究では、日本の地域高齢者を対象とした8年間の前向きコホート研究デザインにより、情報処理速度と総死亡の関連を検討した。

〔方法〕70歳以上の高齢者男性440人と女性371人が本研究に参加した。情報処理速度は符号問題（Digit symbol substitution test)により測定した。符号問題得点を独立変数として、年齢、性別、教育歴、抑うつ傾向、生活習慣病、感覚機能障害、手段的自立、歩行速度、認知障害を共変量として分析に使用した。

〔結果〕追跡期間中に182人（男性133人、女性49人）が死亡した。Coxの比例ハザードモデルの結果、符号問題得点が低いほど死亡リスクが高いことが明らかとなった（第3四分位群におけるハザード比:1.62、95%信頼区間:0.97～2.72; 第2四分位群におけるハザード比:1.73、95%信頼区間：1.05～2.87; 第1四分位群におけるハザード比：2.55、95% 信頼区間：1.51～4.29)。

〔結論〕本研究より、情報処理速度が遅い高齢者は生命予後が不良であることが示唆された。

キーワード：総死亡、認知機能、地域高齢者、情報処理速度
